# Effective viral-mediated lung gene therapy: is airway surface preparation necessary?

**DOI:** 10.1038/s41434-022-00332-7

**Published:** 2022-03-29

**Authors:** Alexandra McCarron, Patricia Cmielewski, Victoria Drysdale, David Parsons, Martin Donnelley

**Affiliations:** 1 Adelaide Medical School, The University of Adelaide, Adelaide, SA Australia; 2 Robinson Research Institute, The University of Adelaide, Adelaide, SA Australia; 3 Department of Respiratory and Sleep Medicine, Women’s and Children’s Hospital, North Adelaide, SA Australia

**Keywords:** Respiratory tract diseases, Genetic transduction

## Abstract

Gene-based therapeutics are actively being pursued for the treatment of lung diseases. While promising advances have been made over the last decades, the absence of clinically available lung-directed genetic therapies highlights the difficulties associated with this effort. Largely, progress has been hindered by the presence of inherent physical and physiological airway barriers that significantly reduce the efficacy of gene transfer. These barriers include surface mucus, mucociliary action, cell-to-cell tight junctions, and the basolateral cell membrane location of viral receptors for many commonly used gene vectors. Accordingly, airway surface preparation methods have been developed to disrupt these barriers, creating a more conducive environment for gene uptake into the target airway cells. The two major approaches have been chemical and physical methods. Both have proven effective for increasing viral-mediated gene transfer pre-clinically, although with variable effect depending on the specific strategy employed. While such methods have been explored extensively in experimental settings, they have not been used clinically. This review covers the airway surface preparation strategies reported in the literature, the advantages and disadvantages of each method, as well as a discussion about applying this concept in the clinic.

## Introduction

### Genetic therapies for lung disease

Genetic therapies hold great potential for the treatment of a range of inherited and acquired pulmonary diseases. The goal of these therapies is usually to restore function of an absent or defective protein to levels that ameliorate the disease symptoms. A range of modalities are currently being explored and these can be divided into five major categories: (1) gene-addition therapy, (2) mRNA therapy, (3) gene repair, (4) mRNA repair, and (5) cell therapy [1, 2]. All approaches are under active investigation and are at various stages in the developmental pipeline, ranging from pre-clinical testing to clinical trial phases.

The first-conceived genetic treatment option and most extensively investigated approach both pre-clinically and clinically is gene-addition, wherein a correct copy of the relevant gene is delivered to the target cells. More recently, attention has turned to the use of mRNA in therapeutics. Rather than employing DNA, mRNA molecules can be delivered to the airway cells in order to express the desired therapeutic protein [3]. Alternatively, mRNA repair approaches, also known as antisense therapies, can be employed. These involve administering short, single-stranded oligonucleotides to cells to target and repair the abnormal mRNA [4]. Development of precise gene editing tools now enable the potential to repair gene mutations in situ via a range of different strategies [5]. Gene-modified cell therapy involves performing permanent gene-correction on ex vivo patient-derived cells and subsequently transplanting the corrected cells into the airways [6].

To be effective, genetic therapies, irrespective of the modality used, require a vehicle to deliver the genetic payload. Non-viral and virus-derived vectors are under development for this purpose. Both have advantages and disadvantages, and these are partly determined by the target organ and cell population(s). Viral-derived vectors take advantage of evolutionary adaptations that enable highly effective entry into human cells. Non-viral vectors are typically less efficient gene-transfer vehicles, but they are easier to manufacture in large quantities, and have reduced immunogenicity and a lower risk profile. The major categories of viral vectors under development for lung-based genetic therapies include adenoviral vectors (AdVs), adeno-associated vectors (AAVs) and lentiviral vectors (LVs), and these will be the focus of this review article.

### Cystic fibrosis: the Holy Grail for lung gene therapy

Targets for lung-directed gene therapy include genetic disorders such as cystic fibrosis (CF) and alpha-1 antitrypsin deficiency, as well as acquired diseases including chronic obstructive pulmonary disease, asthma, lung cancers and others [7, 8]. CF lung disease has long been targeted for development of an effective gene therapy. Unlike other lung disorders that have a complex aetiology and involve the interaction of multiple genes and environmental factors, CF is a monogenic disorder, and thus it has been considered an ideal candidate for gene therapy. CF patients carry two mutated copies of the CF transmembrane conductance regulator (*CFTR*) gene, which encodes for a protein that acts as an epithelial chloride and bicarbonate channel. Dysfunctional CFTR causes disruption to the ion and water balance across the airways, dehydration of the airway surface, accumulation of viscous mucus, and creation of an environment that is ideal for colonisation by opportunistic pathogens. Over time, CF airways are subjugated to cycles of infection and inflammation, ultimately leading to irreversible structural lung damage [9].

Shortly following discovery of the *CFTR* gene in 1989, a gene therapy for lung disease was eagerly being pursued, and it was thought that one would be readily available in the clinic within a couple of years. Initial in vitro and in vivo pre-clinical studies demonstrated promising proof-of-concept for a *CFTR* gene-addition therapy [10], however, early clinical trials performed in CF patients began to reveal efficacy issues. Approximately 30 years on, a genetic therapy for CF lung disease has not come to fruition, despite significant efforts. While delivery of a gene therapy agent to the lungs is relatively simple due to ease of accessing the airways, the reality is that achieving efficient gene transfer in this organ is difficult. Naturally occurring airway barriers substantially reduce the ability for gene vectors to access the target airway cell types and deliver their genetic payload. Since these early CF clinical trials, the challenge of overcoming physical and physiological airway barriers remains one of the most critical and frequently cited impediments to the development of effective lung-directed genetic therapies.

### Physical and physiological airway barriers restrict effective viral vector mediated gene therapy

**Fig. 1:**
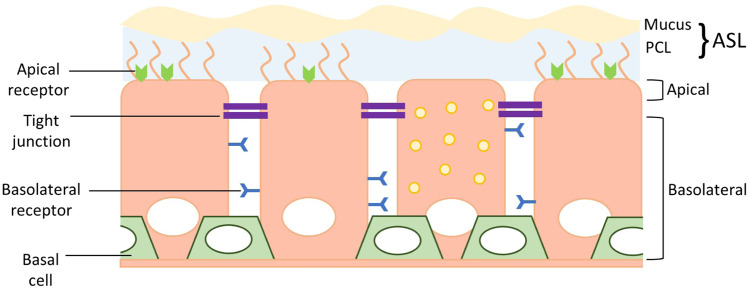
Physical and physiological barriers to viral vector mediated airway gene transfer. The airway epithelium consists of multiple barriers that limit the ability of viral vectors to deliver their transgene to the target cells. Barriers include surface mucus and the action of the mucociliary clearance (MCC); lack of relevant viral vector receptors on the apical membrane; epithelial tight junctions that prevent vector particles accessing basolateral-located receptors; and the deep-lying location of basal stem cells that are not easily accessible via the airway lumen. PCL periciliary liquid, ASL airway surface layer.

The lungs are one of only a few bodily systems exposed to the outside world. Accordingly, evolution has driven the development of features that are designed to protect the airway cells from invasion by airborne pathogens, particulates, and allergens. These barriers can be divided into two major categories: (1) physical/physiological (including cellular) and (2) immunological. While the immune system poses significant issues for gene therapy that must be overcome, this review focuses on physical, physiological and cellular airway barriers to effective airway gene transfer, as these form the first line of defence. The major barriers to viral vector mediated airway gene transfer will be discussed below and are summarised diagrammatically in Fig. 1.

Notably, luminal airway barriers can be circumvented completely by using systemic based delivery systems, such as via intravenous administration. This delivery approach also has the additional benefit of distributing the therapeutic to other organs that may be affected, which is valuable for multi-organ diseases. However, systemic administration may not be suitable for all pulmonary diseases as it tends to target lung endothelial cells and pneumocytes, rather than epithelial cells [11]. For the purposes of this article, we will focus only on airway-directed gene therapies, as these are the most relevant to the topic of this review.

#### Airway surface mucus and mucociliary clearance

Airways are lined with ciliated epithelial cells and an airway surface layer that consists of two components: a mucus layer and the underlying periciliary liquid (PCL). The mucus immobilises inhaled particles and pathogens, while the PCL provides lubrication to facilitate ciliary beating, which results in trapped material being moved from the lungs toward the pharynx for cough clearance or swallowing. The coordinated interaction of these airway components forms a process known as mucociliary clearance (MCC), one of the most critical defences of the airways [12, 13]. In addition to the MCC action, the PCL itself acts as a physical barrier that prevents pathogens from accessing the underlying epithelial cells [8]. While essential for protecting the airways, these defence processes substantially diminish the efficacy of gene-transfer agents. Accordingly, the combined effects of the mucus layer and MCC have been recognised as one of the most significant barriers to effective airway gene therapy.

#### Barrier function of the airway epithelium

If a gene vector can overcome these extracellular obstacles, the airway epithelium is the next hurdle. The epithelium primarily functions as a barrier between the external environment and internal milieu [14]. Tight and adherens junctions are membranous structures located between epithelial cells and contribute significantly to maintaining barrier function. These junctional complexes are critical for regulating the passage of substances across the epithelia and preventing pathogens and foreign bodies from gaining access and causing damage to the subepithelial tissue. Their presence also separates the epithelial layer into two distinct domains, the apical membrane and basolateral membrane [14, 15].

To deliver their genetic cargo to the airway epithelial cells, viral vectors must bind to a complementary receptor expressed on the cell surface, which results in cellular internalisation. Some vectors confer apical entry into airway cells, for example, LV pseudotypes derived from baculovirus (GP64), Sendai virus (F/HN), and influenza (HA) [16], as well as some AAV serotypes including 1, 5, 6, and AAV2.5T [8, 17]. However, many commonly used gene-transfer vectors employ receptors that are located only on the basolateral surface [8]. Examples include AdVs that target the coxsackievirus and adenovirus receptor, certain AAV serotypes (e.g. AAV2) that mediate entry via heparan sulfate proteoglycans, and vesicular-stomatitis-G (VSV-G) pseudotyped LVs that employ the low density lipoprotein receptor, all of which are known to be expressed on the basolateral membrane of the airway cells [18–20]. Epithelial tight junctions prevent viral vectors from gaining access to these deep-lying receptors, resulting in less efficient transduction when administered via the airway lumen.

#### Limited basal cell access via luminal delivery

To achieve long-term therapeutic effect from an airway gene therapy, permanent gene-correction of self-renewing cells will be necessary [21]. Basal cells are a primary stem cell type within the conducting airways and drive epithelial homeostasis, as well as regeneration following injury [22]. Basal cells are anchored to the base of the epithelial layer (basal lamina) and are not in direct contact with the airway lumen. Therefore, while targeting basal cells may be essential for long-term gene expression, viral vector access to these cells is limited.

#### Impact of lung disease state

Pre-clinical development for gene therapies is typically performed in vitro or in non-diseased animal models, and thus does not consider the impact of lung disease state on gene-transfer efficacy. Pathophysiological disease processes including mucus hyperproduction, chronic infection, and inflammation create additional barriers that gene vectors must circumvent. Accordingly, the disease state needs to be considered when developing an effective lung-directed gene therapy.

CF mucus is characteristically thick and adhesive, making it difficult to clear from the airways, creating a trap for inhaled particles [23]. In retrospect, the poor efficacy of early AdV and AAV gene therapy clinical trials in CF patients can be partly attributed to low-level transduction by inhaled vectors due to the presence of mucus and infection [24–26]. Following these unsuccessful CF gene therapy clinical trials, studies have been designed to directly examine the impact of mucus, infection and inflammation on gene transfer.

AdVs and AAVs were unable to effectively penetrate sputum samples collected from CF patients and showed substantially reduced diffusion rates [24, 25, 27]. Given this, it is likely that delivery of gene vectors to the luminal airway surface results in a significant proportion of particles becoming trapped within the mucus layer and cleared via MCC or cough clearance before they have the chance to access the airway epithelial cells. Additionally, advanced CF lung disease can result in regional mucus plugging of the small airways and subsequent air-flow obstruction [23]. In this scenario, a gene therapy formulation delivered as an aerosol or liquid would not be able to effectively access and treat these blocked airways.

Infection and local inflammation are also common in many lung diseases, yet only a handful of studies have investigated the impact of these factors on gene-transfer efficacy. In one study employing a mouse model of induced *Pseudomonas aeruginosa* (*P. aeruginosa*) infection, the effect on gene-transfer was variable depending on the delivery vehicle employed. LV vector mediated gene transfer was not impacted by the presence of infection, while some non-viral DNA carriers (lipofectamine and polyethylenimine) exhibited a significant reduction in transfection ability [28]. In a separate study, infection with common CF respiratory bacterial species (*Bordetella bronchiseptica* or *P. aeruginosa*) negatively impacted AAV vector transduction in mice [26]. The presence of *P. aeruginosa* induced bronchopulmonary inflammation in mice has also been found to reduce AdV vector mediated gene transfer [29].

Further work is needed to fully understand the impact of disease state on airway gene transfer. Assessing the performance of individual gene vectors under disease conditions will be necessary as the biochemical and physical properties of the vector will be significant factors in their success. Until recently, this research was hindered by lack of a suitable animal model with human-like CF lung disease. However, newer models that develop infection and muco-obstruction of the airways, including CF ferret and pig models, may aid in facilitating these studies [30–32]. Ultimately, a lung-targeted genetic therapy will have the greatest efficacy and safety in lungs with a low or absent burden of disease, therefore treatment early in life is the most desirable approach, and has the additional benefit of potentially halting further lung disease progression [27].

#### Overcoming airway barriers

Limited ability to perform effective airway gene transfer has led to the development of novel vector engineering approaches to enhance delivery and uptake into the target cells. Improved airway cell tropism can be achieved by optimising the chosen vector pseudotype for LVs [33, 34] or via capsid engineering for AdVs and AAVs [35]. Similarly, peptides or ligands can be added to the vector surface to target specific cellular receptors [36], or to confer enhanced mucus-penetrating capacity [37]. Altering the surface charge of the vector can increase transduction efficacy [38], while other modifications to surface properties can reduce immunogenicity, for example, the addition of polymers (e.g. PEGylation) can shield particles from the immune system [36].

An alternative strategy to increase gene transfer, and the focus of this review article, is the use of techniques that prepare the airways for gene transfer, making the cells more receptive to transduction.

## Airway surface preparation techniques

Depending on their nature, airway surface preparation techniques can produce a range of biological effects. Described (and hypothesised) effects include removal/dislodgment of airway mucus, impairment of MCC action including deciliation of cells, disruption of cell–cell tight-junction integrity, and sometimes, the removal of surface epithelial cells. These techniques transiently modulate physical and physiological airway barriers, increase vector residence time, as well as enable access to basolateral-located receptors and airway basal cells [39].

Airway surface preparation techniques that have been reported in the literature can be divided into chemical and physical strategies. This section will review and summarise the airway conditioning methods that have been commonly employed in conjunction with viral vector mediated gene transfer. Based on the available evidence, we will comment on the advantages and disadvantages of each approach and speculate on the best options to pursue.

### Chemical conditioning

Chemical-based conditioning is the most frequently reported airway surface preparation method. A range of chemicals with varying properties have been explored for this purpose, including both liquids and gas (Table 1). In some cases, it has been found that the conditioning agent cannot be combined directly with the vector due to significant loss of vector viability. To overcome this, two separate administrations are required—one to deliver the conditioning compound and then a second to deliver the vector once the chemical effect has taken place and the compound is cleared from the airway surface. This increases procedure complexity and is disadvantageous as the distribution of the conditioning agent and viral vector can be variable or mismatched [40]. Importantly, conditioning compounds tend to produce biological effects that are transient, enabling improved gene transfer while minimising the potential for long-lasting impact on the lungs.

**Table 1:** Summary of chemical conditioning methods used in vivo with viral vector mediated airway gene transfer.

Compound	Concentration	Species	Route of vector delivery	Viral vector type	References
LPC	0.1–2%	Mouse, rat	Nasal	LV, HD-AdV	[43–49, 57, 62, 106]
	0.01–1%	Mouse, rat, ferret, rabbit, sheep, pig, marmoset, baboon	Lung	LV, HD-AdV, piggyBac/AdV	[34, 50–56, 58–64]
PDOC	0.1–1%	Mouse	Nasal	AdV, LV	[45, 66]
C10	30–50 mM	Mouse	Lung	AdV	[38, 74]
EGTA	3–400 mM	Mouse, rabbit	Lung	AdV, HD-AdV, AAV, LV, retroviral	[38, 74, 75, 78–80]
PFC	100%	Mouse, rat, macaque	Lung	AdV, AAV	[84, 86–89, 107]
SO_2_	500 ppm	Mouse	Nasal and lung	LV, retroviral	[90, 91]

*mM* millimolar, *ppm* parts per million.

Surfactants were one of the first known compounds to be used as airway conditioning agents and were hypothesised to work by enhancing the uniformity of pulmonary gene transfer. Survanta® (modified bovine pulmonary surfactant) co-delivered with an AdV vector successfully improved airway gene expression in vivo, demonstrating early proof-of-concept [41, 42]. Since these initial studies, the field has favoured the use of synthetic fatty acid-derived surfactants, including lysophosphatidylcholine (LPC), polidocanol (PDOC), and sodium caprate (C10).

#### Lysophosphatidylcholine (LPC)

LPC is one of the most extensively studied airway conditioning agents for viral vector mediated gene transfer. LPC is a natural component of pulmonary surfactant, and when applied to the airways, the histological effects are concentration dependent. High concentrations result in more overt consequences including loss of cilia, lifting or removal of surface cells, and in some cases, stripping of the epithelial layer [43]. LPC also possesses tight-junction opening properties. Transepithelial potential difference measures demonstrated a depolarisation response following nasal LPC administration in mice, indicating a loss of tight-junction barrier function [43]. LPC may also have mucolytic properties and reduce ciliary-beat frequency, both attributes that increase vector residence time [39, 44]. The concentration, volume and timing of LPC administration is dependent on the type of gene vector to be delivered and the target region of the airway.

LPC has been essential for producing LV VSV-G mediated gene transfer in the nasal airways of mice [44–48] and rats [49], provided it was delivered 1 h prior to LV administration. LPC delivered to the lung airways also enhances LV VSV-G vectors with varying degrees of efficacy in a range of animal species including mice [34, 50, 51], rats [52], ferrets [53], sheep [50] and the marmoset [54, 55]. LPC has also been employed with LV vectors pseudotyped with envelope proteins that target apically-located receptors including GP64 [44] and HA [34].

Helper dependent AdV (HD-AdV) is a robust vector particle that can be formulated in LPC (0.01–0.1%), allowing for one-step administration that produced extensive reporter gene transduction after aerosolisation to the lungs of rabbits [56]. Repeated administration of LPC and HD-AdV vector to mouse lungs produced high reporter gene transduction in the conducting airways [57], as did its use in the lungs of baboons [58, 59]. Furthermore, this formulation could be successfully redosed [60]. Efficient LPC and HD-AdV vector transduction was also produced in the conducting airways of pigs [61, 62], and in newborn ferrets [63]. More recently, aerosolisation of LPC and a piggyBac/AdV vector into newborn pigs resulted in strong airway gene transfer [64].

LPC enables successful airway gene transfer using a range of viral vectors and animal species, with its use continuing to be routine in animal studies. The extensive pre-clinical use of LPC (including studies in non-human primate species) suggests a favourable safety profile, though this has not been examined directly. Based on the current evidence, LPC appears to provide variable enhancement effects, highlighting the need to optimise the concentration, volume and timing interval for each individual application.

#### Polidocanol (PDOC)

Other fatty-acid surfactants have also been explored, but less comprehensively than LPC. Polidocanol (PDOC) is a synthetic non-ionic detergent and has previously been used clinically as a locally injectable sclerosing agent for varicose vein treatment, where concentrations up to 3% were found to be well tolerated [65]. PDOC has been shown to improve in vivo airway gene transfer levels. Low PDOC concentrations (0.1%) applied to the nasal airways increases epithelial permeability, in the absence of visible histological changes [66]. PDOC concentrations from 0.1 to 1% substantially increased transduction in the nasal epithelium of mice when used in conjunction with an AdV vector [66] or VSV-G pseudotyped LV vector [45]. In the latter study however, improvements in gene transfer from PDOC conditioning were found to be modest when directly compared to LPC [45].

In more recent times the use of PDOC for facilitation of viral-mediated airway gene transfer has fallen out of favour. Instead, PDOC has been employed in the lungs for other experimental purposes. Application of higher concentrations of PDOC (typically 2%) to the airways of rodents can remove the surface epithelium, while leaving the basal cell layer relatively intact [67]. The ability to remove surface cells is a property that makes PDOC useful for investigating stem cell behaviour and regeneration of the airway epithelium following gene transfer with integrating LV vectors [68, 69]. Transient PDOC-induced lung injury has also been employed prior to delivery of cells, with its use found to enhance retention and engraftment of transplanted cells [67, 70, 71].

#### Sodium caprate (C_10_)

Sodium caprate, also referred to as C_10_, is the sodium salt of the medium-chain fatty-acid capric acid. Sodium caprate has been used clinically to enhance drug permeability across the intestines, and was a component of an approved rectal suppository [72]. Like other fatty-acid derived surfactants used for airway conditioning, sodium caprate has airway tight-junction opening properties, reducing transepithelial resistance when applied to human airway epithelial cultures in vitro. This property is proposed to enable vectors improved access to the basolateral compartment and appropriate viral receptors [73].

Direct formulation of AdV vectors with sodium caprate produces complete loss of vector viability; therefore, two separate administrations are necessary [38, 74]. Sodium caprate increases AdV vector mediated gene transfer to human airway epithelial cultures in vitro and mouse airway epithelium in vivo [38, 73]. In mice, a combined formulation of sodium caprate and EGTA (see below) was applied to the airways prior to AdV vector delivery, however, this did not further increase transduction levels when compared to sodium caprate alone [38]. A similar compound, sodium laurate (C_12_), also enhanced AdV gene transfer [74], but has been investigated less than C_10_.

Application of sodium caprate to the airways of mice induced mild histopathological changes and an increase in airway responsiveness, indicating low-level toxic effects in the lungs [74]. While an effective compound at enhancing viral vector mediated gene transfer, it has not been employed in research applications in more recent times, potentially due to adoption of other fatty-acid conditioning compounds such as LPC that have more favourable characteristics, including more comprehensive investigation under a range of experimental conditions and the ability for co-delivery with some viral vectors.

#### EGTA

Several groups have reported EGTA (ethylene glycol-bis(beta-aminoethyl ether)-N,N,N′,N′-tetraacetic acid) enhances viral vector mediated airway gene transfer. Unlike the fatty-acid derived compounds, EGTA is a calcium-chelating agent that reduces the intracellular concentration of calcium ions to disrupt calcium-dependent formation and stabilisation of tight-junction protein complexes [8, 75]. As would be expected, EGTA application reduces transepithelial resistance, consistent with increased tight-junction permeability, both in cultured human airway epithelial cells in vitro, and in human nasal epithelium in vivo [75].

EGTA conditioning has enhanced airway gene transfer for AAVs, AdVs (including HD-AdVs), LVs and retroviruses [75–79]. Successful retroviral-, lentiviral- and adenoviral-mediated gene transfer to rabbit tracheal epithelium was produced when EGTA conditioning preceded gene delivery, while limited gene transfer was present in the absence of conditioning [75, 79]. Similar improvements with EGTA conditioning for an AdV vector were observed in mice [80], while in vitro studies using human CF airway cultures produced restoration of CFTR function following co-administration of VSV-G LV and EGTA [79]. EDTA, a related compound, has also been examined, but appears to be less effective than EGTA at disrupting airway tight junctions [80].

EGTA offers certain benefits as an airway conditioning agent for use in a clinical setting. Unlike other conditioning agents, EGTA can be formulated with viral vectors (including LVs), allowing for one-step delivery to airways [74, 75, 79]. The effects of EGTA on the junctional complexes are also rapid and reversible [75]. Other calcium chelators such as EDTA are used already for clinical indications including intravenous chelation therapy for lead poisoning [81]. EDTA has also been explored for its antibacterial properties in the lung. In multiple clinical studies, CF patients with *P. aeruginosa* infection received nebulisation of EDTA and a concomitant antibiotic. In these studies, inhaled EDTA resulted in no harmful effects or adverse events [82, 83]. However, assessments of EGTA toxicity performed in mice have noted inflammatory effects in the lungs [74]. Moreover, EGTA appears to be less potent than other conditioning agents, with one study revealing that sodium caprate was more effective than EGTA at enhancing AdV vector transduction in mouse lower airways upon direct comparison [38].

#### Perfluorochemical

Perfluorochemicals (PFCs) are non-toxic substances that consist of chemically inert fluorinated carbon chains. PFC liquid has characteristics that are suited to use in the lungs. It is highly soluble in O_2_ and CO_2_, and has a high density and low surface tension, allowing it to distribute throughout the conducting airways and alveoli [84, 85]. Due to these properties, PFC liquid is proposed to act as a carrier to enable more efficient distribution of vector particles throughout the airways. Delivery of PFC liquid to the airways also induces transient opening of tight junctions, providing vectors improved access to basolateral receptors [86, 87]. Other hypothesised effects of PFC administration include displacement of airway surface mucins and fluid. There is also evidence that the compound interferes with vector phagocytosis by alveolar macrophages, thus increasing the proportion of active viral particles that successfully reach the airway epithelial cells [86].

Use of PFC liquid prior to vector administration improved AdV and AAV vector mediated gene transfer to the airways of rodents [84, 86, 88] and non-human primates [89]. Inhalation of nebulised PFC vapour has also been explored as a simpler and more clinically appealing technique compared to liquid-based delivery. Nebulisation of PFC vapour similarly enhances AdV and AAV mediated gene expression in the airway epithelium of mice and macaques, respectively [87].

PFC has properties that are consistent with a good clinical safety profile. It is not metabolised by the kidneys or liver, and is eliminated by evaporation during exhalation or transpiration through the skin [85]. Non-human primates produced no obvious adverse effects from delivery of liquid or nebulised PFC to the airways [87, 89]. PFC has also proven effective at increasing gene transfer among a range of animal species with various AdV and AAV-based vectors. However, interest in the use of PFC for airway conditioning has declined, with few publications employing this approach in the last decade. It is not apparent why this is the case, but more general concerns regarding the translatability of airway preparation approaches may have contributed. Furthermore, the mechanisms of PFC action are yet to be fully elucidated, particularly in the case of nebulised formulations, which offer the same gene-transfer improvements as the liquid delivery, but do not appear to increase tight-junction permeability [87].

#### Sulphur dioxide inhalation

One gas, sulphur dioxide (SO_2_), has also been explored for enhancing viral vector mediated gene transfer. While delivery of fluid can result in non-homogeneous effects, gases provide the benefit of uniform distribution throughout the airways, particularly when two separate administrations are required for the conditioning compound and viral vector. There are two proposed mechanisms that underpin the gene transfer enhancement effects from SO_2_ inhalation: (1) direct injury and denuding of the surface epithelium due to luminal cell death and sloughing following SO_2_, and (2) increased paracellular permeability in areas of less severe injury, allowing viral vectors access to basolateral receptors and basal cells [90].

A handful of studies have explored the use of SO_2_ for airway conditioning. In one study, mice receiving SO_2_ inhalation-induced injury followed by delivery of a murine leukaemia retroviral vector demonstrated significant improvement in tracheal cell transduction, while those without SO_2_ injury had no observable gene expression [91]. Similarly, a LV VSV-G pseudotyped vector demonstrated substantial gene expression in the nasal and tracheal epithelium of mice and rats when delivered following SO_2_ inhalation, while gene transfer did not occur in the absence of SO_2_ conditioning [90].

SO_2_ inhalation successfully enhances airway gene transfer in rodent models and offers delivery advantages over liquid formulations. While this work highlighted the feasibility of using a gas for airway surface preparation, there has not been any further studies in this area. Lack of interest is likely due to the inability to translate this approach to the clinic, particularly given the severe airway injury produced and toxic effects associated with SO_2_ inhalation [92].

### Physical perturbation

**Fig. 2:**
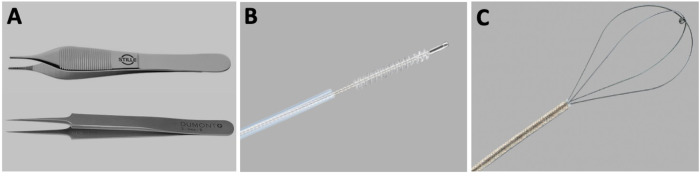
Examples of physical perturbation devices. **A** Blunt and fine forceps, (**B**) bronchial cytology brush (Cook Medical), and (**C**) flexible wire basket (NCircle^®^, Cook Medical).

Physical perturbation of the airways involves the use of a method or device (examples depicted in Fig. 2) to remove cells or otherwise disrupt the integrity of the airway epithelium. Disturbance of the epithelium aids transduction, potentially by revealing basolateral viral receptors [79], or dislodging the poorly transducible surface epithelial cells to expose underlying basal cells that may be more susceptible to transduction [93, 94]. Physical perturbation may also offer other benefits, including removal of the protective mucus layer or disruption of local mucociliary transport to facilitate more direct access to epithelial cells and improved residence time, but these effects have not been investigated.

High levels of in vivo airway gene transfer are commonly reported in regions inadvertently abraded with delivery instrumentation, such as an endotracheal tube or a bronchoscope [50, 94]. Moreover, the first airway gene therapy trial performed in the nasal epithelium of CF patients using an AdV vector retrospectively concluded that viral transduction was likely facilitated by airway damage caused by the delivery method [10]. While incidental findings, they provided early evidence of the effectiveness of physical perturbation strategies. Studies have since been performed to directly investigate the gene transfer enhancement effects of physical approaches, however, the techniques employed were relatively crude. The use of a pipette tip to scratch an epithelial sheet before apical delivery of a LV vector enhanced ex vivo transduction in damaged area [79]. Physical perturbation using forceps to externally compress or scrape excised CF human trachea also increased transduction in abraded regions [93, 94].

In a previous investigation by Pickles et al. the intercartilaginous regions of mouse tracheas were externally compressed with forceps and AdV vector delivered via a tracheostomy, resulting in distinct lines of gene expression in the compressed regions [94]. However, while this approach appeared to enhance AdV gene transfer, only low levels of intermittent gene expression were achieved when it was used in conjunction with a LV vector [90]. Another study used forceps inserted through a tracheostomy tube to remove linear regions of epithelial cells in mouse trachea. Areas exposed to perturbation prior to AdV vector delivery showed greater reporter gene expression than unperturbed sections [93]. Perturbation of rabbit tracheas and bronchi with a bronchial cytology brush via an endotracheal tube resulted in an increase in retroviral vector transduction [95]. Recently, a fine, flexible wire basket that conforms to the airway lumen was used to physically perturb rat tracheas prior to administration of a LV vector, resulting in a 1000-fold increase in the area of LacZ staining over the unperturbed controls [96].

Physical perturbation successfully enhances viral-mediated airway gene transfer, however, the gene expression produced tends to be non-uniform across the tissue. This is likely because the gene-transfer enhancement effects localise only to the regions where the device is applied, therefore achieving widespread gene transfer throughout all airways with this method may be challenging. However, the limited local effects produced by physical perturbation may also be an advantage, as this will prevent extensive damage to the airways. Furthermore, targeted or successive treatments to localised lung regions could be enabled with this approach. Variable efficacy of physical perturbation has been noted in animal studies, which could be due to the use of relatively crude techniques and instruments, however, the more recent use of a flexible wire basket in rat airways indicates the ability to refine these methods [96]. Moreover, incorporating visualisation with a bronchoscope in the future may facilitate targeted perturbation to the lower conducting airways [52], which is the primary target for lung gene therapies, rather than the trachea.

Clinical translation of this approach may be more challenging than chemical methods due to procedural complexity and the need for controlled and precise techniques. Importantly, the lung has an extensive ability to respond to injury and regenerate lost or damaged cells [97], as shown in the above studies demonstrating successful repair of the airway epithelium following perturbation [94, 96]. This regenerative capacity means that inducing localised, controlled damage via physical perturbation techniques is unlikely to have long-lasting impacts on the airways. However, increased cell turnover post-perturbation may also result in loss of gene-expressing cells, particularly if basal cells are poorly targeted and non-integrating vectors are used. Physical perturbation methods will require in-depth safety examination for clinical translation, but unlike the use of chemicals, physical approaches have the advantage that they will not require characterisation of toxicity and metabolism profiles.

## Clinical translation of airway surface preparation techniques

An important notion that arises when employing conditioning methods for airway gene therapy is the ability to translate these techniques to the clinic. Ultimately, should these methods be restricted to the realms of experimental investigations, or is there value in actively pursuing selected techniques for clinical development?

Conditioning methods act to intentionally disrupt naturally occurring protective airway barriers, which raises concerns, particularly when they are employed in a lung with existing infection, inflammation, and tissue damage. In particular, there are concerns that disrupting the epithelial integrity could enhance the leakage of bacterial products and inflammatory mediators into the submucosa, resulting in further damage to the lungs [39]. Moreover, these methods could allow antibiotic-resistant bacteria to gain access to systemic circulation, particularly in cases of advanced lung disease [6]. Disturbing the integrity of cellular tight junctions can also have other potential consequences. Junctional complexes are known to serve as signalling platforms for regulation of gene expression, cell proliferation and differentiation. Accordingly, conditioning processes that disrupt tight-junction integrity may interfere with normal repair and differentiation of the airway epithelium [14], however, further work is needed to explore this phenomenon, particularly in diseased lung environments.

For chemical methods, understanding the toxicity and metabolism profiles of the compound is critical. While investigators have explored some safety aspects of conditioning compounds in experimental settings, more extensive assessment is necessary for use in humans. In addition to this, the delivery procedure must be considered. Two-step protocols where conditioning is performed prior to gene vector delivery are more complicated than one-step methods, and for chemical approaches, nebulised delivery of conditioning agents is preferable to fluid administration in clinical settings. For physical perturbation, the procedure will require patient sedation to enable successive deployment of the device to the airway branches.

There are currently no clinically available airway gene therapies, and protocols that are nearing early phase clinical trials do not include an airway surface preparation step [98]. In the absence of any precedence, we need to examine therapeutic strategies used in other organs. One concept of a similar procedural premise is the use of pre-transplant conditioning for hematopoietic stem cell transplantation. For many decades, stem cell transplantations were attempted with little success, until it was realised that damage was necessary to create space in the recipient’s bone marrow for the engraftment and expansion of stem cells. Conditioning typically includes a combination of chemotherapy and radiotherapy, processes that are known to be toxic and produce adverse effects [99], but are the accepted consequences for achieving effective transplantation. Indeed, chemotherapeutic drugs such as doxorubicin are being actively explored as agents for augmenting airway gene transfer. Doxorubicin, a proteasome inhibitor, has been shown to enhance airway cell transduction of several AAV serotypes by facilitating translocation of the vector to the nucleus [100].

Looking more closely at the lungs, there are many clinically used procedures that are considered invasive and produce some level of airway damage. For example, the use of whole lung lavage is an effective treatment for alveolar proteinosis, and more recently, silicosis [101, 102]. While the protocol is not consistent between specialised treatment centres, the procedure can involve endotracheal intubation of each lung and the repeated filling and draining of up to 50 l of saline per lung [101]. Damage to regions of the airway epithelium is also considered an accepted consequence of clinical bronchoscopies. Flexible bronchoscopy and bronchoalveolar lavage are standard procedures in the clinical care of paediatric and adult patients with lung diseases. These procedures are safe and well tolerated, despite being performed in infected and inflamed airways [103]. Another lung-based procedure, bronchial thermoplasty, is a novel asthma treatment that acts to reduce airway smooth muscle mass and subsequently, airway resistance. The technique involves delivering controlled thermal energy to the bronchial airways via a bronchoscope under direct visual guidance [104]. Follow-up of a cohort of bronchial thermoplasty patients 10 years post-procedure concluded that the technique had an acceptable safety profile [105].

Examining these routinely used procedures suggests that there is sound rationale for the application of airway surface preparation methods in the clinic to enhance the effectiveness of gene transfer. However, the risk versus benefit ratio needs to be considered. If high levels of gene transfer can be achieved using these strategies, then significant clinical benefit is expected. Furthermore, improved access to airway stem cells facilitated by conditioning techniques could enable gene correction of this self-renewing population of cells, providing potential for long-term gene expression and therapeutic effect.

## Concluding remarks

Airway-delivered genetic therapies continue to show poor efficacy due to the presence of natural airway barriers. Early gene therapy clinical trials performed in CF patients using AdV and AAV-based vectors demonstrated safety and proof-of-concept, but many of these trials failed to meet their primary endpoints and patients demonstrated poor gene expression [10], highlighting the sub-therapeutic levels of correction obtained when attempts are not made to actively mitigate these barriers. Airway surface preparation techniques offer an effective approach for improving gene transfer, and act to temporarily modulate physical and physiological airway barriers to enable gene vectors improved access to the target epithelial cell types. Evidence from in vivo studies describing both chemical and physical techniques demonstrate that airway surface conditioning can produce highly effective vector transduction and subsequent gene expression. The effect can be substantial, as shown by some vectors failing to achieve gene transfer in the absence of airway surface preparation. Airway conditioning approaches are yet to be employed clinically, and further safety data is necessary before this can be contemplated. While there are risks to consider with these approaches, producing highly effective gene transduction is likely to confer significant clinical benefit. Importantly, if stem cells are successfully transduced, the patient may receive long-term clinical benefit without the need for frequent therapy re-administration.

## References

[1] Alton EW, Boyd AC, Davies JC, Gill DR, Griesenbach U, Harrison PT (2016). Genetic medicines for CF: hype versus reality. Pediatr Pulmonol.

[2] Allan KM, Farrow N, Donnelley M, Jaffe A, Waters SA. Treatment of cystic fibrosis: from gene- to cell-based therapies. Front Pharmacol. 2021;12:639475. 10.3389/fphar.2021.639475 PMC8007963 33796025

[3] Sahu I, Haque AKMA, Weidensee B, Weinmann P, Kormann MSD (2019). Recent developments in mRNA-based protein supplementation therapy to target lung diseases. Mol Ther.

[4] Martinovich KM, Shaw NC, Kicic A, Schultz A, Fletcher S, Wilton SD (2018). The potential of antisense oligonucleotide therapies for inherited childhood lung diseases. Mol Cell Pediatr.

[5] Ensinck M, Mottais A, Detry C, Leal T, Carlon MS. On the corner of models and cure: gene editing in cystic fibrosis. Front Pharmacol. 2021;12:662110. 10.3389/fphar.2021.662110 PMC8111007 33986686

[6] King NE, Suzuki S, Barillà C, Hawkins FJ, Randell SH, Reynolds SD (2020). Correction of airway stem cells: genome editing approaches for the treatment of cystic fibrosis. Hum Gene Ther.

[7] Albelda SM, Wiewrodt R, Zuckerman JB (2000). Gene therapy for lung disease: hype or hope?. Ann Intern Med.

[8] Kim N, Duncan GA, Hanes J, Suk JS (2016). Barriers to inhaled gene therapy of obstructive lung diseases: a review. J Control Release.

[9] Turcios NL (2020). Cystic fibrosis lung disease: an overview. Respir Care.

[10] Cooney AL, McCray PB Jr, Sinn PL. Cystic fibrosis gene therapy: looking back, looking forward. Genes. 2018;9:538. 10.3390/genes9110538 PMC6266271 30405068

[11] Gautam A, Waldrep CJ, Densmore CL (2002). Delivery systems for pulmonary gene therapy. Am J Respir Med.

[12] Bustamante-Marin XM, Ostrowski LE (2017). Cilia and mucociliary clearance. Cold Spring Harb Perspect Biol.

[13] Donnelley M, Gardner M, Morgan K, Parsons D. Chapter 8—Non-absorptive clearance from airways. In: Kassinos S, Bäckman P, Conway J, Hickey AJ, editors. Inhaled medicines. Academic Press; 2021. pp 197–223.

[14] Ganesan S, Comstock AT, Sajjan US (2013). Barrier function of airway tract epithelium. Tissue Barriers.

[15] Soini Y (2011). Claudins in lung diseases. Respir Res.

[16] Griesenbach U, Inoue M, Meng C, Farley R, Chan M, Newman NK (2012). Assessment of F/HN-pseudotyped lentivirus as a clinically relevant vector for lung gene therapy. Am J Respir Crit Care Med.

[17] Excoffon KJDA, Koerber JT, Dickey DD, Murtha M, Keshavjee S, Kaspar BK (2009). Directed evolution of adeno-associated virus to an infectious respiratory virus. Proc Natl Acad Sci USA.

[18] Coyne CB, Bergelson JM (2005). CAR: a virus receptor within the tight junction. Adv Drug Deliv Rev.

[19] O’Donnell J, Taylor KA, Chapman MS (2009). Adeno-associated virus-2 and its primary cellular receptor—Cryo-EM structure of a heparin complex. Virology..

[20] Finkelshtein D, Werman A, Novick D, Barak S, Rubinstein M (2013). LDL receptor and its family members serve as the cellular receptors for vesicular stomatitis virus. Proc Natl Acad Sci USA.

[21] Cooney AL, Thurman AL, McCray PB, Pezzulo AA, Sinn PL. Lentiviral vectors transduce lung stem cells without disrupting plasticity. bioRxiv. 2020. 10.1016/j.omtn.2021.06.010 PMC8379527 34458011

[22] Rock JR, Randell SH, Hogan BL (2010). Airway basal stem cells: a perspective on their roles in epithelial homeostasis and remodeling. Dis Model Mech.

[23] Boucher RC (2019). Muco-obstructive lung diseases. N Engl J Med.

[24] Hida K, Lai SK, Suk JS, Won SY, Boyle MP, Hanes J (2011). Common gene therapy viral vectors do not efficiently penetrate sputum from cystic fibrosis patients. PLoS ONE.

[25] Schuster BS, Kim AJ, Kays JC, Kanzawa MM, Guggino WB, Boyle MP (2014). Overcoming the cystic fibrosis sputum barrier to leading adeno-associated virus gene therapy vectors. Mol Ther.

[26] Myint M, Limberis MP, Bell P, Somanathan S, Haczku A, Wilson JM (2014). In vivo evaluation of adeno-associated virus gene transfer in airways of mice with acute or chronic respiratory infection. Hum Gene Ther.

[27] Stern M, Caplen NJ, Browning JE, Griesenbach U, Sorgi F, Huang L (1998). The effect of mucolytic agents on gene transfer across a CF sputum barrier in vitro. Gene Ther.

[28] Rejman J, De Fino I, Paroni M, Bragonzi A, Demeester J, De Smedt S (2010). Impact of chronic pulmonary infection with Pseudomonas aeruginosa on transfection mediated by viral and nonviral vectors. Hum Gene Ther.

[29] van Heeckeren A, Ferkol T, Tosi M (1998). Effects of bronchopulmonary inflammation induced by Pseudomonas aeruginosa on adenovirus-mediated gene transfer to airway epithelial cells in mice. Gene Ther.

[30] Sun X, Olivier AK, Liang B, Yi Y, Sui H, Evans TIA (2013). Lung phenotype of juvenile and adult cystic fibrosis transmembrane conductance regulator–knockout ferrets. Am J Respir Cell Mol Biol.

[31] Stoltz DA, Meyerholz DK, Pezzulo AA, Ramachandran S, Rogan MP, Davis GJ (2010). Cystic fibrosis pigs develop lung disease and exhibit defective bacterial eradication at birth. Sci Transl Med.

[32] McCarron A, Donnelley M, Parsons D (2018). Airway disease phenotypes in animal models of cystic fibrosis. Respir Res.

[33] Duvergé A, Negroni M. Pseudotyping lentiviral vectors: when the clothes make the virus. Viruses. 2020;12:1311. 10.3390/v12111311 PMC7697029 33207797

[34] Carpentieri C, Farrow N, Cmielewski P, Rout-Pitt N, McCarron A, Knight E (2021). The effects of conditioning and lentiviral vector pseudotype on short- and long-term airway reporter gene expression in mice. Hum Gene Ther.

[35] Li C, Samulski RJ (2020). Engineering adeno-associated virus vectors for gene therapy. Nat Rev Genet.

[36] Capasso C, Hirvinen M, Cerullo V (2013). Beyond gene delivery: strategies to engineer the surfaces of viral vectors. Biomedicines..

[37] Leal J, Peng X, Liu X, Arasappan D, Wylie DC, Schwartz SH (2020). Peptides as surface coatings of nanoparticles that penetrate human cystic fibrosis sputum and uniformly distribute in vivo following pulmonary delivery. J Control Release.

[38] Gregory LG, Harbottle RP, Lawrence L, Knapton HJ, Themis M, Coutelle C (2003). Enhancement of adenovirus-mediated gene transfer to the airways by DEAE dextran and sodium caprate in vivo. Mol Ther.

[39] Castellani S, Conese M (2010). Lentiviral vectors and cystic fibrosis gene therapy. Viruses..

[40] Flotte TR, Ng P, Dylla DE, McCray PB, Wang G, Kolls JK (2007). Viral vector-mediated and cell-based therapies for treatment of cystic fibrosis. Mol Ther.

[41] Jobe AH, Ueda T, Whitsett JA, Trapnell BC, Ikegami M (1996). Surfactant enhances adenovirus-mediated gene expression in rabbit lungs. Gene Ther.

[42] Katkin JP, Husser RC, Langston C, Welty SE (1997). Exogenous surfactant enhances the delivery of recombinant adenoviral vectors to the lung. Hum Gene Ther.

[43] Cmielewski P, Anson DS, Parsons DW (2010). Lysophosphatidylcholine as an adjuvant for lentiviral vector mediated gene transfer to airway epithelium: effect of acyl chain length. Respir Res.

[44] Kremer KL, Dunning KR, Parsons DW, Anson DS (2007). Gene delivery to airway epithelial cells in vivo: a direct comparison of apical and basolateral transduction strategies using pseudotyped lentivirus vectors. J Gene Med.

[45] Limberis M, Anson DS, Fuller M, Parsons DW (2002). Recovery of airway cystic fibrosis transmembrane conductance regulator function in mice with cystic fibrosis after single-dose lentivirus-mediated gene transfer. Hum Gene Ther.

[46] Stocker AG, Kremer KL, Koldej R, Miller DS, Anson DS, Parsons DW (2009). Single-dose lentiviral gene transfer for lifetime airway gene expression. J Gene Med.

[47] Cmielewski P, Donnelley M, Parsons DW (2014). Long-term therapeutic and reporter gene expression in lentiviral vector treated cystic fibrosis mice. J Gene Med.

[48] Cmielewski P, Delhove J, Donnelley M, Parsons D (2021). Assessment of lentiviral vector mediated CFTR correction in mice using an improved rapid in vivo nasal potential difference measurement protocol. Front Pharmacol.

[49] Reyne N, Cmielewski P, McCarron A, Delhove J, Parsons D, Donnelley M (2021). Single-dose lentiviral mediated gene therapy recovers CFTR function in cystic fibrosis knockout rats. Front Pharmacol.

[50] Liu C, Wong E, Miller D, Smith G, Anson D, Parsons D (2010). Lentiviral airway gene transfer in lungs of mice and sheep: successes and challenges. J Gene Med.

[51] Cmielewski P, Farrow N, Devereux S, Parsons D, Donnelley M (2017). Gene therapy for cystic fibrosis: improved delivery techniques and conditioning with lysophosphatidylcholine enhance lentiviral gene transfer in mouse lung airways. Exp Lung Res.

[52] McIntyre C, Donnelley M, Rout-Pitt N, Parsons D (2018). Lobe-specific gene vector delivery to rat lungs using a miniature bronchoscope. Hum Gene Ther Methods.

[53] Cmielewski P, Farrow N, Donnelley M, McIntyre C, Penny-Dimri J, Kuchel T (2014). Transduction of ferret airway epithelia using a pre-treatment and lentiviral gene vector. BMC Pulm Med.

[54] Farrow N, Miller D, Cmielewski P, Donnelley M, Bright R, Parsons DW (2013). Airway gene transfer in a non-human primate: lentiviral gene expression in marmoset lungs. Sci Rep.

[55] Farrow N, Cmielewski P, Delhove J, Rout-Pitt N, Vaughan L, Kuchel T (2021). Towards human translation of lentiviral airway gene delivery for cystic fibrosis: a one-month CFTR and reporter gene study in marmosets. Hum Gene Ther.

[56] Koehler DR, Frndova H, Leung K, Louca E, Palmer D, Ng P (2005). Aerosol delivery of an enhanced helper-dependent adenovirus formulation to rabbit lung using an intratracheal catheter. J Gene Med.

[57] Koehler DR, Martin B, Corey M, Palmer D, Ng P, Tanswell AK (2006). Readministration of helper-dependent adenovirus to mouse lung. Gene Ther.

[58] Hiatt P, Brunetti-Pierri N, Koehler D, McConnell R, Katkin J, Palmer D (2005). Aerosol delivery of helper-dependent adenoviral vector into nonhuman primate lungs results in high efficiency pulmonary transduction with minimal toxicity. Mol Ther.

[59] Hiatt P, Brunetti-Pierri N, McConnell R, Palmer D, Katkin J, Dimmock D (2006). Bronchoscope-guided, targeted lobar aersolization of HDAd into the lungs of nonhuman primate results in exceedingly high pulmonary transduction uniformally throughout the entire lung with negligible toxicity. Mol Ther.

[60] Hiatt P, Brunetti-Pierri N, McConnell R, Palmer DJ, Vetrini F, Grove N (2008). Pulmonary transduction in nonhuman primates by HDAd: duration of transgene expression and vector re-administration. Mol Ther.

[61] Cao H, Machuca TN, Yeung JC, Wu J, Du K, Duan C (2013). Efficient gene delivery to pig airway epithelia and submucosal glands using helper-dependent adenoviral vectors. Mol Ther Nucleic Acids.

[62] Cao H, Ouyang H, Grasemann H, Bartlett C, Du K, Duan R (2018). Transducing airway basal cells with a helper-dependent adenoviral vector for lung gene therapy. Hum Gene Ther.

[63] Yan Z, Stewart ZA, Sinn PL, Olsen JC, Hu J, McCray PB (2015). Ferret and pig models of cystic fibrosis: prospects and promise for gene therapy. Hum Gene Ther Clin Dev.

[64] Cooney AL, Singh BK, Loza LM, Thornell IM, Hippee CE, Powers LS (2018). Widespread airway distribution and short-term phenotypic correction of cystic fibrosis pigs following aerosol delivery of piggyBac/adenovirus. Nucleic Acids Res.

[65] Eckmann DM (2009). Polidocanol for endovenous microfoam sclerosant therapy. Expert Opin Investig Drugs.

[66] Parsons DW, Grubb BR, Johnson LG, Boucher RC (1998). Enhanced in vivo airway gene transfer via transient modification of host barrier properties with a surface-active agent. Hum Gene Ther.

[67] Gui L, Qian H, Rocco KA, Grecu L, Niklason LE (2015). Efficient intratracheal delivery of airway epithelial cells in mice and pigs. Am J Physiol Lung Cell Mol Physiol.

[68] Mitomo K, Griesenbach U, Inoue M, Somerton L, Meng C, Akiba E (2010). Toward gene therapy for cystic fibrosis using a lentivirus pseudotyped with Sendai virus envelopes. Mol Ther.

[69] Farrow N, Donnelley M, Cmielewski P, Roscioli E, Rout-Pitt N, McIntyre C (2018). Role of basal cells in producing persistent lentivirus-mediated airway gene expression. Hum Gene Ther.

[70] Leblond A-L, Naud P, Forest V, Gourden C, Sagan C, Romefort B (2009). Developing cell therapy techniques for respiratory disease: intratracheal delivery of genetically engineered stem cells in a murine model of airway injury. Hum Gene Ther.

[71] Farrow N, Cmielewski P, Donnelley M, Rout-Pitt N, Moodley Y, Bertoncello I (2018). Epithelial disruption: a new paradigm enabling human airway stem cell transplantation. Stem Cell Res Ther.

[72] Twarog C, Fattah S, Heade J, Maher S, Fattal E, Brayden DJ. Intestinal permeation enhancers for oral delivery of macromolecules: a comparison between salcaprozate sodium (SNAC) and sodium caprate (C10). Pharmaceutics. 2019;11:78. 10.3390/pharmaceutics11020078 PMC6410172 30781867

[73] Coyne CB, Kelly MM, Boucher RC, Johnson LG (2000). Enhanced epithelial gene transfer by modulation of tight junctions with sodium caprate. Am J Respir Cell Mol Biol.

[74] Johnson LG, Vanhook MK, Coyne CB, Haykal-Coates N, Gavett SH (2003). Safety and efficiency of modulating paracellular permeability to enhance airway epithelial gene transfer in vivo. Hum Gene Ther.

[75] Wang G, Zabner J, Deering C, Launspach J, Shao J, Bodner M (2000). Increasing epithelial junction permeability enhances gene transfer to airway epithelia In vivo. Am J Respir Cell Mol Biol.

[76] Duan D, Yue Y, Yan Z, McCray PB, Engelhardt JF (1998). Polarity influences the efficiency of recombinant adenoassociated virus infection in differentiated airway epithelia. Hum Gene Ther.

[77] Walters RW, van’t Hof W, Yi SM, Schroth MK, Zabner J, Crystal RG (2001). Apical localization of the coxsackie-adenovirus receptor by glycosyl-phosphatidylinositol modification is sufficient for adenovirus-mediated gene transfer through the apical surface of human airway epithelia. J Virol.

[78] Koehler DR, Sajjan U, Chow Y-H, Martin B, Kent G, Tanswell AK (2003). Protection of Cftr knockout mice from acute lung infection by a helper-dependent adenoviral vector expressing Cftr in airway epithelia. Proc Natl Acad Sci USA.

[79] Wang G, Slepushkin V, Zabner J, Keshavjee S, Johnston JC, Sauter SL (1999). Feline immunodeficiency virus vectors persistently transduce nondividing airway epithelia and correct the cystic fibrosis defect. J Clin Investig.

[80] Chu Q, St George JA, Lukason M, Cheng SH, Scheule RK, Eastman SJ (2001). EGTA enhancement of adenovirus-mediated gene transfer to mouse tracheal epithelium in vivo. Hum Gene Ther.

[81] George T, Brady MF. Ethylenediaminetetraacetic Acid (EDTA) [Updated 2021 Jul 18]. In: StatPearls [Internet]. Treasure Island (FL): StatPearls Publishing; 2022 Jan. Available from: https://www.ncbi.nlm.nih.gov/books/NBK565883/. 33351441

[82] Brown J, Mellis CM, Wood RE (1985). Edetate sodium aerosol in pseudomonas lung infection in cystic fibrosis. Am J Dis Child.

[83] Puvvadi R, Mikkelsen H, McCahon L, Grogan S, Ditcham W, Reid DW (2021). Role of Tris-CaEDTA as an adjuvant with nebulised tobramycin in cystic fibrosis patients with Pseudomonas aeruginosa lung infections: a randomised controlled trial. J Cyst Fibros.

[84] Weiss DJ, Strandjord TP, Jackson JC, Clark JG, Liggitt D (1999). Perfluorochemical liquid-enhanced adenoviral vector distribution and expression in lungs of spontaneously breathing rodents. Exp Lung Res.

[85] Tawfic QA, Kausalya R (2011). Liquid ventilation. Oman Med J..

[86] Weiss DJ, Bonneau L, Allen JM, Miller AD, Halbert CL (2000). Perfluorochemical liquid enhances adeno-associated virus-mediated transgene expression in lungs. Mol Ther.

[87] Beckett T, Bonneau L, Howard A, Blanchard J, Borda J, Weiner DJ (2012). Inhalation of nebulized perfluorochemical enhances recombinant adenovirus and adeno-associated virus-mediated gene expression in lung epithelium. Hum Gene Ther Methods.

[88] Li JT, Bonneau LA, Zimmerman JJ, Weiss DJ (2007). Perfluorochemical (PFC) liquid enhances recombinant adenovirus vector-mediated viral interleukin-10 (AdvIL-10) expression in rodent lung. J Inflamm.

[89] Weiss DJ, Baskin GB, Shean MK, Blanchard JL, Kolls JK (2002). Use of perflubron to enhance lung gene expression: safety and initial efficacy studies in non-human primates. Mol Ther.

[90] Johnson LG, Olsen JC, Naldini L, Boucher RC (2000). Pseudotyped human lentiviral vector-mediated gene transfer to airway epithelia in vivo. Gene Ther.

[91] Johnson LG, Mewshaw JP, Ni H, Friedmann T, Boucher RC, Olsen JC (1998). Effect of host modification and age on airway epithelial gene transfer mediated by a murine leukemia virus-derived vector. J Virol.

[92] Bast CB, Koller L, Woodall G. Sulfur dioxide. Acute exposure guideline levels. Washington, DC, USA: United States: National Academy Press; 2010.

[93] Grubb BR, Pickles RJ, Ye H, Yankaskas JR, Vick RN, Engelhardt JF (1994). Inefficient gene transfer by adenovirus vector to cystic fibrosis airway epithelia of mice and humans. Nature..

[94] Pickles RJ, Barker PM, Ye H, Boucher RC (1996). Efficient adenovirus-mediated gene transfer to basal but not columnar cells of cartilaginous airway epithelia. Hum Gene Ther.

[95] Halbert CL, Aitken ML, Miller AD (1996). Retroviral vectors efficiently transduce basal and secretory airway epithelial cells in vitro resulting in persistent gene expression in organotypic culture. Hum Gene Ther.

[96] McCarron A, Farrow N, Cmielewski P, Knight E, Donnelley M, Parsons D. Breaching the delivery barrier: chemical and physical airway epithelium disruption strategies for enhancing lentiviral-mediated gene therapy. Front Pharmacol. 2021;12:669635. 10.3389/fphar.2021.669635 PMC8107471 33981244

[97] Kotton DN, Morrisey EE (2014). Lung regeneration: mechanisms, applications and emerging stem cell populations. Nat Med.

[98] Alton EWFW, Beekman JM, Boyd AC, Brand J, Carlon MS, Connolly MM, et al. Preparation for a first-in-man lentivirus trial in patients with cystic fibrosis. Thorax. 2017;72:137. 10.1136/thoraxjnl-2016-208406 PMC5284333 27852956

[99] Zulu S, Kenyon M. Principles of conditioning therapy and cell infusion. In: Kenyon M, Babic A, editors. The European Blood and Marrow Transplantation Textbook for Nurses: Under the Auspices of EBMT. Cham: Springer International Publishing; 2018. pp 89–96. 31314221

[100] Yan Z, Sun X, Feng Z, Li G, Fisher JT, Stewart ZA (2015). Optimization of recombinant adeno-associated virus-mediated expression for large transgenes, using a synthetic promoter and tandem array enhancers. Hum Gene Ther.

[101] Trapnell BC, Nakata K, Bonella F, Campo I, Griese M, Hamilton J (2019). Pulmonary alveolar proteinosis. Nat Rev Dis Primers.

[102] Chambers DC, Apte SH, Deller D, Masel PJ, Jones CM, Newbigin K (2021). Radiological outcomes of whole lung lavage for artificial stone-associated silicosis. Respirology..

[103] Du Rand IA, Blaikley J, Booton R, Chaudhuri N, Gupta V, Khalid S (2013). British Thoracic Society guideline for diagnostic flexible bronchoscopy in adults: accredited by NICE. Thorax..

[104] Laxmanan B, Hogarth DK (2015). Bronchial thermoplasty in asthma: current perspectives. J Asthma Allergy.

[105] Chaudhuri R, Rubin A, Sumino K, Lapa e Silva JR, Niven R, Siddiqui S (2021). Safety and effectiveness of bronchial thermoplasty after 10 years in patients with persistent asthma (BT10+): a follow-up of three randomised controlled trials. Lancet Respir Med.

[106] Cao H, Yang T, Li XF, Wu J, Duan C, Coates AL (2011). Readministration of helper-dependent adenoviral vectors to mouse airway mediated via transient immunosuppression. Gene Ther.

[107] Weiss DJ, Mutlu GM, Bonneau L, Mendez M, Wang Y, Dumasius V (2002). Comparison of surfactant and perfluorochemical liquid enhanced adenovirus-mediated gene transfer in normal rat lung. Mol Ther.

